# Automated Assessment of Cerebral Arterial Perforator Function on 7T MRI

**DOI:** 10.1002/jmri.27304

**Published:** 2020-08-18

**Authors:** Tine Arts, Jeroen C.W. Siero, Geert Jan Biessels, Jaco J.M. Zwanenburg

**Affiliations:** ^1^ Department of Radiology, Centre for Image Sciences University Medical Center Utrecht Utrecht The Netherlands; ^2^ Spinoza Centre for Neuroimaging Amsterdam The Netherlands; ^3^ Department of Neurology University Medical Center Utrecht Utrecht The Netherlands

**Keywords:** blood‐flow velocity, blood‐flow pulsatility, cerebral arterial perforators, 7 Tesla MRI, ghosting artifacts

## Abstract

**Background:**

Blood flow velocity and pulsatility of small cerebral perforating arteries can be measured using 7T quantitative 2D phase contrast (PC) MRI. However, ghosting artifacts arising from subject movement and pulsating large arteries cause false positives when applying a previously published perforator detection method.

**Purpose:**

To develop a robust, automated method to exclude perforators located in ghosting artifacts.

**Study Type:**

Retrospective.

**Subjects:**

Fifteen patients with vascular cognitive impairment or carotid occlusive disease and 10 healthy controls.

**Field Strength/Sequence:**

7T/cardiac‐gated 2D PC MRI.

**Assessment:**

Perforators were automatically excluded from ghosting regions, which were defined as bands in the phase‐encoding direction of large arteries. As reference, perforators were manually excluded by two raters (T.A., J.J.M.Z.), based on perforator location with respect to visible ghosting artifacts. The performance of both censoring methods was assessed for the number of (N_included_), mean velocity (V_mean_), and pulsatility index (PI) of the included perforators.

**Statistical Tests:**

For within‐method comparisons, inter‐ and intrarater reliability were assessed for the manual method, and test–retest reliability was assessed for both methods from repeated 2D PC scans (without repositioning). Intraclass correlation coefficients (ICCs) and their 95% confidence intervals (CIs) were determined for N_included_, V_mean_, and PI for all within‐method comparisons. The ICC to compare between the two methods was determined with the use of both (test–retest) scans using a multilevel nonlinear mixed model.

**Results:**

The automated censoring method showed a moderate to good ICC (95% CI) vs. manual censoring for N_included_ (0.73 [0.58–0.87]) and V_mean_ (0.90 [0.84–0.96]), and a moderate ICC for PI (0.57 [0.37–0.76]). The test–retest reliability of the manual censoring method was considerably lower than the interrater and intrarater reliability, indicating that scanner noise dominates the uncertainty of the analysis.

**Data Conclusion:**

The proposed automated censoring method can reliably exclude small perforators affected by ghosting artifacts.

**Level of Evidence:**

3.

**Technical Efficacy Stage:**

1.

CEREBRAL SMALL VESSEL DISEASE (SVD) is a widespread condition that affects the small arteries, arterioles, venules, and capillaries of the brain and is the main cause of cognitive decline and stroke.^1^ Also, it is involved in approximately half of all dementia cases.^2^ However, insight into the exact processes that lead to cerebral small vessel disease is limited, and this hampers the development of effective treatment. Therefore, measuring the flow properties of the small cerebral perforating arteries may improve our understanding of cerebral hemodynamics^3^ and may be of use for treatment assessment.

It is possible to measure the hemodynamics of the cerebral perforating arteries using a cardiac‐gated 2D phase contrast (2D PC) magnetic resonance imaging (MRI) sequence at 7 Tesla (T).^4^, ^5^ This method utilizes the increased signal‐to‐noise ratio (SNR) of 7T MRI and the fact that the relatively low blood flow velocities in these small perforators (100–300 μm) allow for a very low readout bandwidth, which further enhances the SNR to a level that makes the flow velocity measureable in the perforators. The method yields the mean velocity (V_mean_) during the cardiac cycle and the pulsatility index (PI) of the blood flow in the perforators of deep white matter (WM), specifically the centrum semi‐ovale (CSO). In this brain region vessels are located that penetrate deep into the brain tissue, branch relatively little, and are low in density. This makes the CSO vulnerable in the case of diseased vessels such as in SVDs and is therefore a region of interest to assess perforator flow.^6^, ^7^, ^8^, ^9^

Previous results showed good repeatability in these metrics in older subjects^4^; however, the processing procedure is hampered by ghosting artifacts arising from subject movement and pulsations from large cerebral arteries.^5^ Ghosts in the phase‐encoding direction (Fig. 1) reflect phase inconsistencies in the multishot acquisition, which are due to subject movement and intershot variation in blood flow pulsations of large cerebral vessels in combination with the low encoding velocity.^10^, ^11^ Ghosting artifacts are visible in data from healthy subjects but are more prominent in data from elderly patients, who tend to move more during the examination.^12^

**FIGURE 1:**
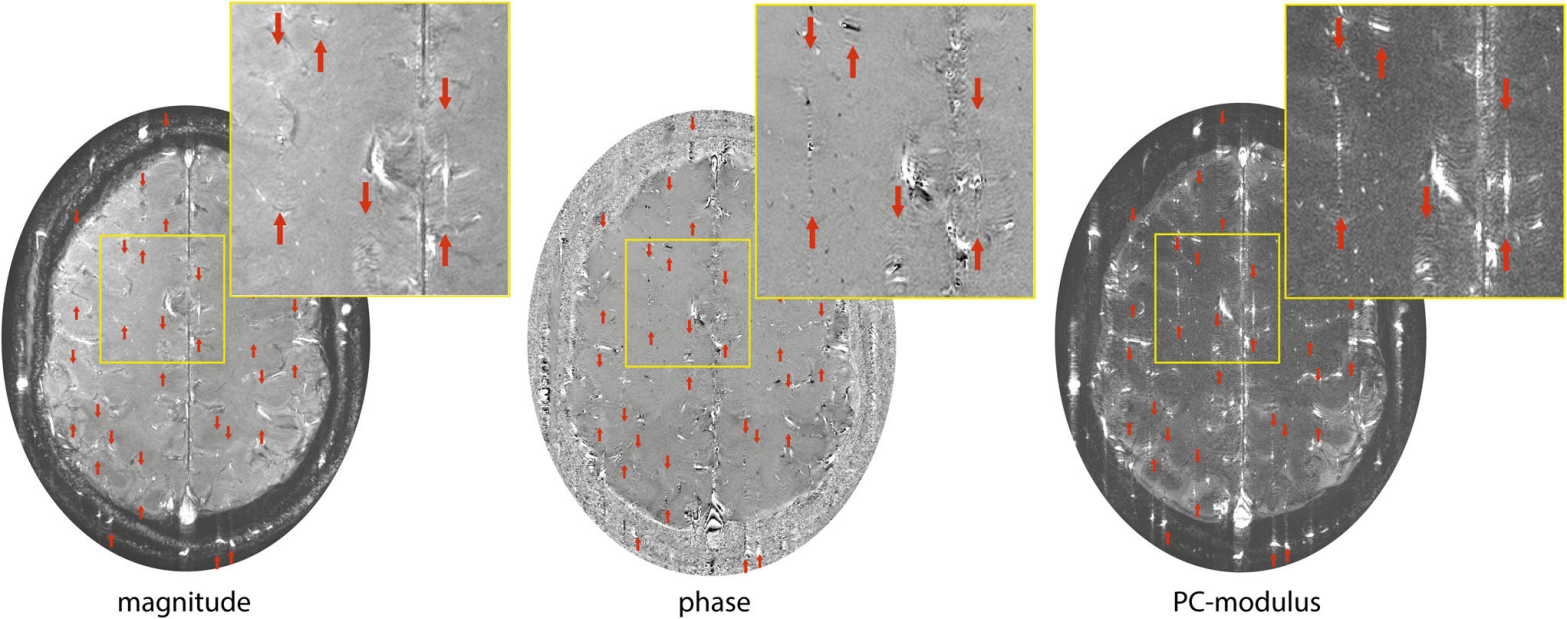
Magnitude, phase, and phase contrast (PC)‐modulus images. Ghosting artifacts (between the arrowheads) are apparent in the phase‐encoding direction, mainly in the phase and PC‐modulus images.

To reduce false‐positive perforator detection, perforators initially detected in these artifact regions should be excluded. Currently, this censoring step is done manually.^5^ While manual censoring is manageable for small datasets, it becomes time‐consuming for large datasets, particularly when data from elderly patients is involved. Besides, manual procedures make the censoring process subjective and reduce the precision in calculated measures describing perforator hemodynamics. Therefore, the main goal of this work was to develop a robust, automated censoring method for fast exclusion of small perforating arteries in artifact regions in the CSO.

## Materials and Methods

### *Image Acquisition*

Our proposed method was evaluated using data from an ongoing study that was approved by our local Institutional Review Board, and all subjects provided written informed consent. The mixed subject group consisted of 20 patients with either vascular cognitive impairment (Mini Mental State Examination ≥20) or carotid occlusive disease (according to the most recent clinical guidelines) and 10 healthy controls (recruited through advertising leaflets in the hospital or spouses of patients). Subjects were required to be aged over 50 and were excluded in case of a contraindication for MRI. Of the 20 patients, five were excluded from analysis due to poor scan quality caused by excessive movement during scanning (*n =* 2), severe patient discomfort (*n =* 1), scanner issues (*n =* 1), or a noisy peripheral pulse unit signal resulting in poor triggering for retrospective gating (*n =* 1). Of the healthy controls, no one was excluded (aged 68 ± 8 years, mean ± standard deviation). Of the 15 included patients (aged 69 ± 8 years, mean ± standard deviation), five were diagnosed with cognitive impairment and 10 with carotid occlusive disease.

**FIGURE 2:**
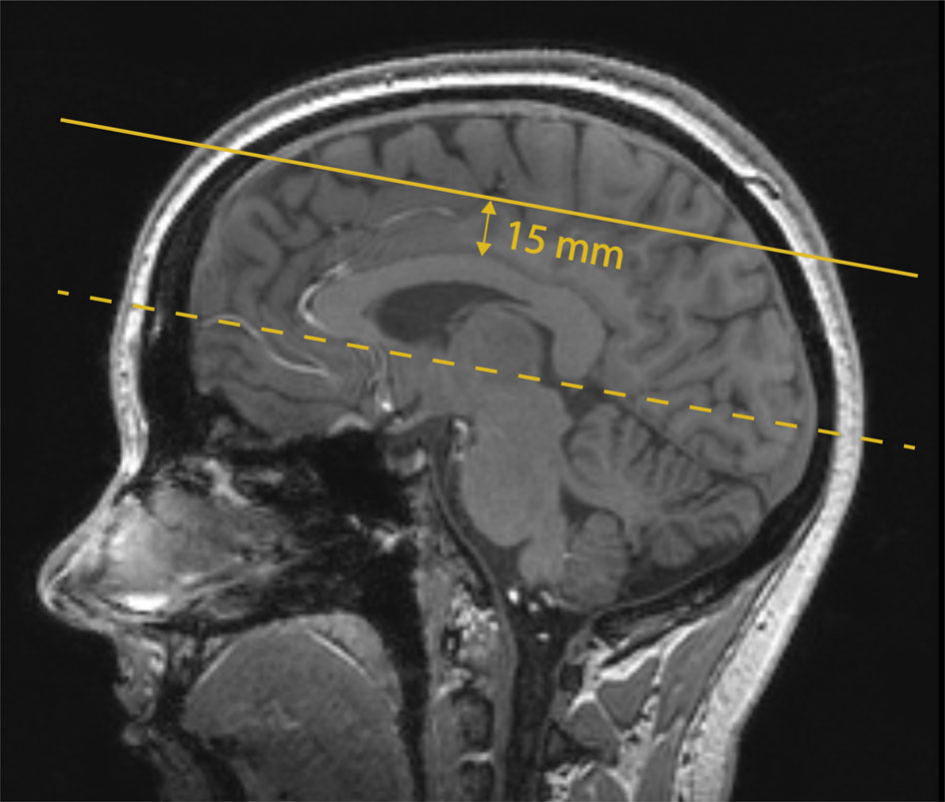
Planning of the 2D PC slice in the centrum semi‐ovale projected on the T_1_‐weighted image. The dashed line is aligned with the bottom of the corpus callosum, indicating the angle of the slice. The slice is then located 15 mm superior to the top of the corpus callosum (solid line).

Images were acquired with a 7T Philips Achieva MRI system (Philips Healthcare, Best, the Netherlands) with a 32‐channel receive head coil (Nova Medical, Wilmington, MA). A 3D T_1_‐weighted image (T_1_WI) was acquired for WM segmentation with an isotropic resolution of 1.0 mm^3^ and whole brain coverage.^4^ The CSO was scanned using a previously described retrospectively gated 2D PC sequence.^4^ Scan parameters included the following: field of view = 250 × 250 mm^2^, acquired voxel size = 0.3 × 0.3 × 2.0 mm^3^ (interpolated to 0.2 × 0.2 mm^2^ in‐plane resolution), flip angle = 50–90° (increasing flip angle across the slice using tilted optimized nonsaturating excitation), sensitivity encoding = 1.5, velocity encoding = 4 cm/s, and 14 reconstructed cardiac phases (for one participant 12 cardiac phases were acquired due to a lower heart rate during scanning). Scan duration was 4 minutes for a heart rate of 78 bpm (range: 3 minutes 10 seconds to 4 minutes 35 seconds). The repetition time (TR) and echo time depend on the angle of the planned slice and ranged from 28.8–30.2 msec and 16.6–17.7 msec, respectively. For every cardiac timepoint, two velocity encoding cycles were acquired (ie, two phase‐encoding steps per heartbeat or turbo field‐echo factor = 2), leading to an acquired temporal resolution of 4*TR (~118 msec). The scan was planned manually, with a target slice location 15 mm superior to the top of the corpus callosum (see Fig. 2). To minimize motion, cushions were placed beside the head of the scanned subject during all scans. For test–retest assessment, this 2D PC scan was performed twice in succession without repositioning.

### *Automated Small Perforator Selection*

WM masks of the CSO were generated using the 3D T_1_WI with a white matter probability threshold of 0.75 (SPM12, Wellcome Trust Centre for Neuroimaging, London, UK). For each 2D PC scan (ie, two per subject), the mask was manually corrected in case of improper segmentation due to either failure of the segmentation algorithm (four subjects, ie, eight masks) or considerable subject shift between the T_1_WI and the 2D PC scans (four subjects, ie, eight masks). Only the central WM pixels were included that were more than 80 pixels away from the outside contour of the 2D PC brain slice mask (a 2D PC pixel equals 0.2 mm).

An initial, uncensored perforator selection was performed within the WM mask using a previously published method^4^, ^13^ that includes the following steps. First, the background was phase‐corrected to make the mean velocity of tissue 0 cm/sec by median filtering the velocity map and subtracting it from the velocity map of each cardiac timepoint. Then the velocity SNR was calculated from an estimation of the magnitude SNR to enable consistent selection of vessels with significant flow. Further, the two‐sided 95% velocity confidence intervals (CIs) were estimated for V_mean_, which is consistent with a statistical significance of 0.05. Finally, perforators were identified based on significant V_mean_. All voxels inside the WM mask without 0 cm/sec within their CI of V_mean_ were considered significant, and every group of neighboring significant voxels was defined as belonging to the same perforator. Only perforators with downward flow, thus negative V_mean_, were included; and for every group, the voxel with the highest absolute V_mean_ was taken as the perforator.

### *Manual Censoring*

**FIGURE 3:**
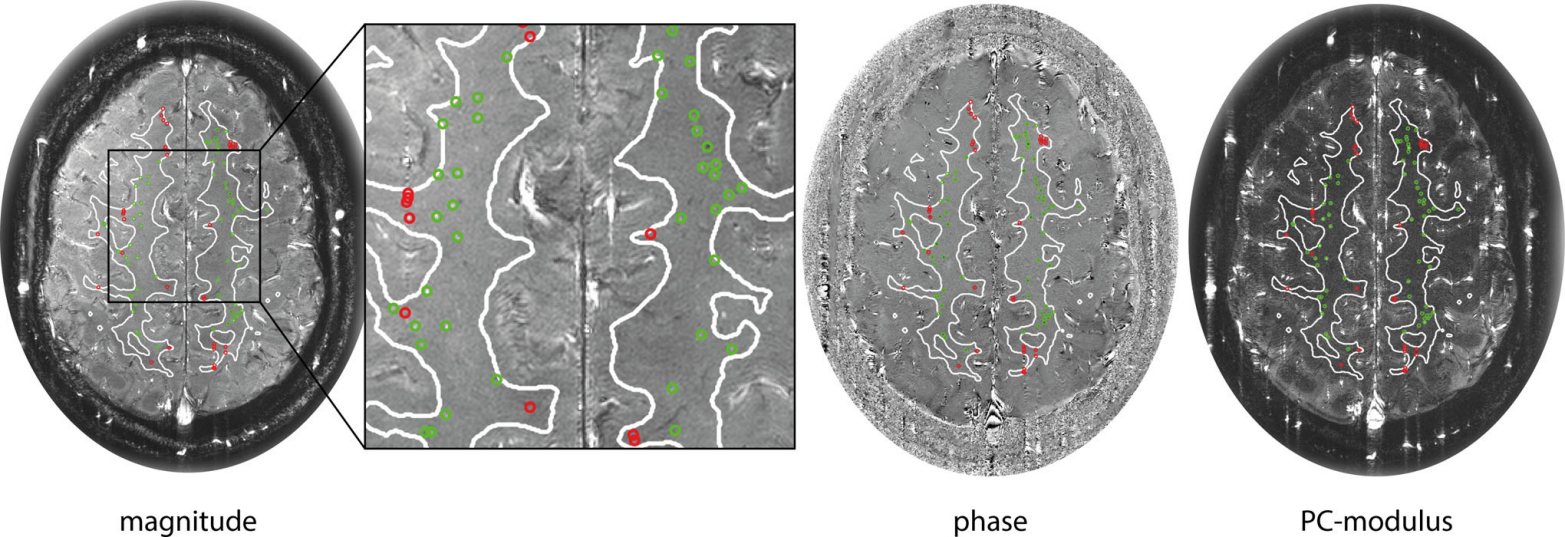
Example of the manual censoring method. Detected perforators located inside ghosting artifacts are circled in red, perforators located outside ghosting artifacts are circled in green, projected on the magnitude, phase, and PC‐modulus image.

Two raters (Rater 1: T.A., 2 years experience; Rater 2: J.J.M.Z., 14 years experience) visually determined the presence and extent of ghosting paths from both the 2D PC magnitude and phase images and manually selected perforators located in ghosting regions for exclusion (Fig. 3). The intrarater reliability was assessed by performing manual exclusions by Rater 1 at two timepoints, 2 months apart. Interrater reliability was determined by having Rater 2 perform the manual exclusions at one timepoint.

### *Automated Censoring*

**FIGURE 4:**
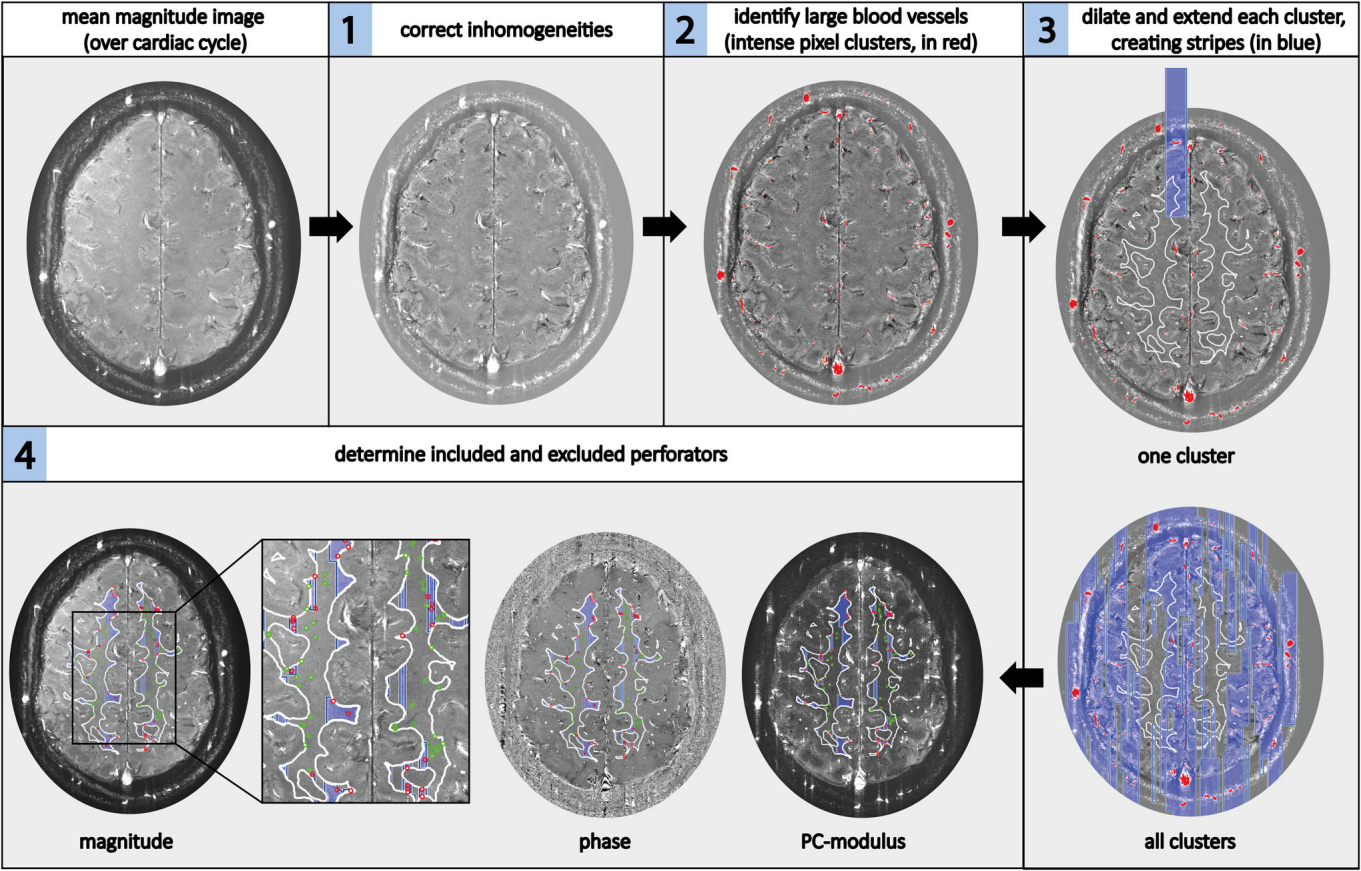
Overview of the automated censoring method. Steps 1–4, as described in the Materials and Methods under the heading Automated Censoring. In step 1, spatial inhomogeneities are corrected. In step 2, the automatically identified large arteries of more than two voxels are shown in red on the detrended and filtered image. In step 3, the vertical stripes created are shown in blue, first for one cluster, then for all clusters. The white matter mask contour is shown in white on the magnitude image. In step 4, the included perforators are circled in green (located outside the stripes) and the excluded perforators are circled in red (located on the stripes) on the magnitude, phase, and PC‐modulus image.

The automated censoring method was applied with the following steps, which are illustrated in Fig. 4:

1. Correct for spatial intensity variation in the mean (over the cardiac cycle) magnitude PC image by applying a median filter (window 70 × 70 pixels) and subtracting the median filtered magnitude image from the original mean magnitude image.
2. Automatically identify large blood vessels by applying a relative intensity threshold on the PC magnitude image. This threshold was defined by sorting the voxels' intensity values from high to low and selecting the top 0.3% of that sorting. Only voxel clusters consisting of more than two voxels were taken into account to not misidentify small perforators as large vessels.
3. Dilate these clusters in the readout direction, and extend them in the phase‐encoding direction (direction in which ghosts occur) to create vertical stripes, partially overlapping the WM mask. Clusters ≥80 voxels were dilated by 2 pixels and extended by 200 pixels in the phase‐encoding direction. Smaller clusters were dilated by 1 pixel and extended by 110 pixels in the phase‐encoding direction.
4. Determine which small perforators are located outside the stripes (included perforators) and which are located on the stripes (excluded perforators).

### *Quantitative Measures*

Both censoring methods resulted in a number of perforators, N_included_, in the WM of the CSO for each subject and scan. For each perforator, an average velocity of the blood was obtained. Averaging over all perforators resulted in a mean blood flow velocity per subject (V_mean_). To calculate the pulsatility index, the perforators' velocity curves were first normalized and subsequently averaged.

The PI was calculated from the resulting mean normalized velocity curve with the following formula:

$$\mathrm{PI}=\left(V_{\max}-V_{\min}\right)/V_{\text{mean}}.$$

Concerning the relative magnitude of the ghosting artifact regions, we evaluated the percentages of how much of the original WM mask is defined as ghosting artifact regions after applying the automated censoring method.

### *Statistical Tests*

A within‐method reliability assessment enabled us to assess the reliability of the automated censoring method and the manual censoring method for N_included_, V_mean_, and PI. Further, a between‐method comparison allowed us to measure the closeness of agreement between the measured values, ie, N_included_, V_mean_, and PI, of the two censoring methods.

#### *WITHIN‐METHOD RELIABILITY ASSESSMENT*

For the automated as well as the manual censoring methods, test–retest reliability of N_included_, V_mean_, and PI was determined on the first compared to the second scan using intraclass correlation coefficients (ICCs) and their 95% CIs derived from a single measurement two‐way mixed‐effects model using the SPSS statistical package (Chicago, IL). Both absolute agreement and consistency were tested. In addition, for the manual censoring method, ICCs and their 95% CIs of intrarater (T.A.) and interrater (T.A. vs. J.J.M.Z.) reliability were determined on the first scan, again using a single measurement two‐way mixed‐effects model with absolute agreement for intrarater comparisons and consistency for interrater comparisons.^14^

An ICC > 0.90 was defined as “excellent,” an ICC between 0.75–0.90 as “good,” an ICC between 0.5 and 0.75 as “moderate,” and an ICC < 0.5 was considered “poor".^14^

#### *BETWEEN‐METHOD COMPARISON*

ICC values were also determined to compare the results of the automated and manual censoring (rater T.A.) methods for the measures N_included,_ V_mean_, and PI. For this comparison, a multilevel statistical analysis was performed using SAS software with the NLMIXED procedure (Non‐Linear MIXED model procedure, SAS v. 9.4, SAS Institute, Cary, NC), comparing the automated and manual censoring method while taking into account the repeated measures, ie, scan 1 and scan 2.

For scan 1 we registered the time needed for rating the perforators for the manual censoring method for all subjects and both raters. These times were then averaged over all subjects and both raters to obtain a mean manual rating time per subject. The automated method was a push‐of‐a‐button method and thus required no timing assessment.

## Results

No significant differences were found between patients and controls for the automated or manual censoring method, for N_included_ (*P* = 0.68; *P* = 0.36), V_mean_ (*P* = 0.22; *P* = 0.41), or PI (*P* = 0.50; *P* = 0.19) using an unpaired Student's *t*‐test where *P* < 0.05 was considered statistically significant. Also, the mean velocity curves over the cardiac cycle (normalized and nonnormalized) did not significantly differ between both groups for both methods. Therefore, patient and control groups were pooled. Analyses separated by group, as well as representative velocity traces of a patient and a control subject with *P*‐values resulting from significance testing per cardiac phase, are included in Supplementary Material A.

### *Within‐Method Reliability Assessment*

Table 1 shows the ICCs and 95% CIs of the automated and manual censoring methods for test–retest reliability assessment, and the ICCs and their 95% CIs for the manual intrarater and interrater reliability assessment. For the automated, as well as the manual censoring method, the ICC values showed good to excellent reliability for N_included_ and V_mean_ for test–retest reliability. PI showed moderate to good reliability. Comparing the reliability of both methods, the automated censoring method had equal or higher ICCs compared to the manual censoring method.^14^ Bland–Altman plots of these results can be found in Supplementary Material B.

**TABLE 1:** Reliability of the Automated and Manual Censoring Methods (ICC [95% CI]) for 25 Subjects

		N_included_	V_mean_	PI
*Test–retest*	*Automated censoring*			
	Consistency	0.90 (0.79–0.96)	0.90 (0.78–0.95)	0.66 (0.36–0.83)
	Absolute agreement	0.91 (0.80–0.96)	0.90 (0.79–0.96)	0.66 (0.37–0.83)
	*Manual censoring, Rater 1*			
	Consistency	0.84 (0.67–0.93)	0.87 (0.74–0.94)	0.50 (0.14–0.74)
	Absolute agreement	0.84 (0.67–0.93)	0.88 (0.74–0.95)	0.48 (0.13–0.73)
*Intrarater*	*Manual censoring, Rater 1* ^a^			
	Absolute agreement	0.99 (0.97–0.99)	0.99 (0.99–1.0)	0.87 (0.73–0.94)
*Interrater*	*Manual censoring Rater 1 vs. Rater 2* ^a^			
	Consistency	0.94 (0.93–0.99)	0.96 (0.92–0.98)	0.88 (0.86–0.97)

ICC = intraclass correlation coefficient; CI = confidence interval; N_included_ = number of included perforators; V_mean_ = mean blood flow velocity during the cardiac cycle; PI = pulsatility index.

^a^ The intrarater and interrater ICCs are based on the first of the repeated phase contrast scans for the manual censoring method.

### *Between‐Method Comparison*

Table 2 shows the outcome measure values for N_included_, V_mean_, and PI for scan 1 and scan 2 and the ICC values and their 95% CIs for the comparison between the automated and manual censoring methods. The ICC (95% CI) values of N_included_ (0.73 [0.58–0.87]), as well as V_mean_ (0.90 [0.84–0.96]), showed moderate to good agreement between the two methods. PI showed moderate agreement (0.57 [0.37–0.76]).^14^

**TABLE 2:** Comparison Between the Automated and Manual Censoring Methods for 25 Subjects

	N_included_ ± SD		V_mean_ (cm/s) ± SD		PI ± SD	
	*Scan 1*	*Scan 2*	*Scan 1*	*Scan 2*	*Scan 1*	*Scan 2*
*Automated censoring*	33 ± 18	33 ± 24	0.71 ± 0.15	0.70 ± 0.13	0.43 ± 0.17	0.45 ± 0.13
*Manual censoring*	38 ± 19	36 ± 21	0.69 ± 0.11	0.69 ± 0.13	0.40 ± 0.14	0.45 ± 0.14
*Multi‐level ICC (95% CI) based on both scans*	0.73 (0.58–0.87)		0.90 (0.84–0.96)		0.57 (0.37–0.76)	

Data are reported as mean ± standard deviation (SD).

ICC = intraclass correlation coefficient; CI = confidence interval; N_included_ = number of included perforators; V_mean_ = mean blood flow velocity during the cardiac cycle; PI = pulsatility index.

After WM segmentation and possible mask adjustments, it took 6.0 ± 1.9 minutes (mean ± standard deviation) per subject to manually select perforators outside ghosting artifacts. The automated censoring method did not cost time.

The WM regions defined as ghosting artifacts by the automated censoring method comprised 65% ± 15% (mean ± standard deviation) of the original WM region (assessed on scan 1).

## Discussion

We have shown that automated censoring of small cerebral perforators affected by ghosting artifacts gives similar values for N_included,_ V_mean_, and PI with equal or improved reliability compared to manual censoring. Because the automated method runs without user input, and thus does not occupy the user, it is faster, saving on average 6 minutes per subject compared to the manual method. Also, the automated method is inherently objective, as it removes any potential rater bias.

Comparison between the automated and manual censoring methods showed moderate to good ICC for N_included_ and V_mean_. PI showed moderate ICC, but the value was similar to the ICC of the test–retest reliability analysis with the automated method. This indicates that a variation in PI between methods is comparable to that between scans and, thus, is dominated by the noise of the measurements rather than by differences in performance between the two analysis methods.

Because the PI calculation is relatively sensitive to noise,^13^ two calculation methods for determining the PI were compared. For the first method, the PIs of all detected perforators were calculated first, after which these PIs were averaged to obtain a single PI for each subject. For the second method, all normalized velocity curves were averaged first, after which the PI was calculated. The second method showed to be the least sensitive to noise and was therefore used in this study. Simulation results for both PI calculation methods can be found in Supplementary Material C.

For manual censoring, the ICC values for the intrarater comparison were excellent and generally higher than the test–retest and interrater ICCs. Additionally, the variation between scans was higher than the variation between raters for all outcome measures. Together, this indicates that the uncertainty is mainly due to the noise in the images.

The automated censoring method showed better or equal test–retest reliability than the manual censoring method for all outcome measures, confirming the objective and consistent nature of the automated method. Although the rater applying the manual censoring method can mostly distinguish between perforators in‐ and outside ghosting paths, the vertical extent of a ghosting path is not always clear, making it difficult to objectively judge perforators in areas where the ghost diminishes. However, for the automated method, varying the areas defined as ghosting regions had little effect on the outcome measures N_included_, V_mean_, and PI (Supplementary Material D). We therefore expect that for the manual censoring method the subjective judgment of where the ghost diminishes also has limited effect on N_included_, V_mean_, and PI.

The mean velocity and pulsatility values reported in this article are in agreement with earlier studies.^4^, ^13^ As expected, the number of perforators included for the V_mean_ and PI calculations, N_included_, is less than in the aforementioned studies because in this study only perforators located outside ghosting artifacts are included. However, the number of included perforators is high enough for reliable PI measurements, as shown by the simulation results in Supplementary Material C.

It is important to note that the method of censoring may affect N_included_, V_mean_, and PI. Therefore, when analyzing small perforator PC MRI data, attention should be paid to what method is used for small perforator censoring and the method must be the same for all included subjects.

This automated method also permits assessment of perforator density, because not only the number of perforators located outside ghosting artifacts is known (N_included_), but the size of the analyzed area is also known, because the automated method distinguishes between WM regions with and without ghosting artifacts. This is not the case for the manual method for which ghosting regions are not quantitatively/spatially defined. For both methods, it is the case that the mask size, and thus also N_included_, highly depends on the severity and amount of the ghosting artifacts present in a PC image. Therefore, comparing N_included_ between groups may introduce some bias.

The presented automated method allows for the measurement of the hemodynamics of small cerebral perforators in an objective and fast manner, which may improve our understanding of the functioning of the cerebral microvasculature and its role in cognitive impairment and dementia.^15^ It is known that vessels become stiffer with aging, reducing the dampening of the pressure wave as blood travels to the perforators. This results in an increased pulse pressure and microvascular remodeling and a decrease in regional autoregulation.^16^, ^17^, ^18^, ^19^ This could damage the capillaries and tissue.^16^ Jefferson et al^20^ found reductions in regional cerebral blood flow (CBF) despite autoregulation being preserved in central arterial stiffening. Because the exact mechanisms involved in cerebral vascular impairment are still unknown, velocity and pulsatility measures of the small cerebral perforators can be of great value to determine how damage to these perforators relates to vascular brain injuries such as microbleeds, infarcts, and decreased WM integrity and shed light on the changes occurring in patients with vascular cognitive impairment.

### *Limitations*

First, the stringency of our automated censoring method depends on parameter settings (the intensity threshold for large vessel identification as well as the length and width of the stripes) and affects how many and which perforators are included. The parameter settings used in this study were chosen empirically to visually match the automatically censored perforators and the manually censored perforators as closely as possible for a subset of the data. However, systematically varying these parameters around the empirically chosen value and assessing the resulting changes showed only little effect on N_included_, V_mean_, and PI (Supplementary Material D).

Second, our automated censoring method still requires a manual processing step: the WM mask, as segmented by SPM12, needs manual adjustments when necessary. If the mask is not properly adjusted, this can lead to false‐positive or false‐negative perforator detection, making the results less accurate. It is particularly important to exclude sulci from the mask to avoid false inclusion of the vessels that run over the cortex. Therefore, a user check and potential corrections in the WM mask remain a required step. Note that this is required for both the manual and the automated censoring methods.

Also, given the subvoxel size of the perforators detected in this study, partial volume averaging is an issue in velocity quantification. We have previously shown that partial volume averaging results in underestimation of flow velocity and overestimation of pulsatility.^13^ Therefore, if changes occur in V_mean_ and PI, we cannot distinguish to what extent this is due to actual changes in velocity or changes in perforator diameter. Nonetheless, changes in velocity and pulsatility do indicate perforator changes, but assumptions regarding vessel diameter are needed for further interpretation.

Additionally, we did not reposition the subject in between the two PC scans, which would have been a more thorough test–retest reliability assessment. However, this was not performed because 1) noise due to planning of the PC slice is very limited compared to thermal and physiological noise, as we have previously shown,^4^ 2) repositioning and replanning would add additional variation between the repeated scans that could possibly mask subtle differences in performance between the analysis methods, and 3) repositioning would make the scanning session substantially longer and increase the burden for the participant.

Finally, subject motion remains a major cause of artifacts in the PC image acquisition used in this study. Our method accounts for ghosting artifacts resulting from nonexcessive motion, but other motion‐related artifacts such as blurring can still result in inaccurate velocity and pulsatility measurements.

Moreover, the number of subjects included in this study was limited, particularly when a comparison between patients and controls would have been the focus. However, for assessing the performance of the censoring methods, we were able to pool the data of the patients and controls, and assess the performance of the methods on a fair number of subjects.

Finally, in this study the manual censoring method was used as a reference method, which is not a gold standard. Up to now, no gold standard exists to distinguish between perforators affected and unaffected by ghosting artifacts.

### *Conclusion*

We have shown that it is feasible to automatically detect small cerebral perforators affected by ghosting artifacts in 2D PC images with a method that is reliable, more objective, and faster compared to manual selection. Our proposed method may provide more accurate quantitative measures of the cerebral microcirculation for the study of small vessel disease.

## Supporting information

Supplementary Materials
