# Supplementary material for: Automated Assessment of Cerebral Arterial Perforator Function on 7T MRI
Source: J Magn Reson Imaging. 2020 Aug 18;53(1):234–41. doi: 10.1002/jmri.27304 (PMC7754489; doi:10.1002/jmri.27304)
Supplement: Supplementary file 1 — Supplementary Materials [file JMRI-53-234-s001.docx]

**Supplementary Material A**

Table A1 shows that no significant differences exist between the patient and control group, for N_included_, V_mean_ as well as PI. Figure A1 shows no significant differences between both groups in the mean velocity curves over the cardiac cycle. In Figure A2 the mean velocity traces of a representative control and patient subject are given including a velocity trace of a cerebral perforator of that subject.

| Table A1: Average values, standard deviations and *p-*values of N_included_, V_mean_ and PI for comparison between the patient group and control group. | | | | | | | | | |
| --- | --- | --- | --- | --- | --- | --- | --- | --- | --- |
|  | N_included_ | | | V_mean_ | | | PI | | |
|  | patients | controls | *p*-  value | patients | controls | *p*-value | patients | controls | *p*-value |
| *Automated censoring* | 31±20 | 35±15 | 0.68 | 0.68±0.12 | 0.75±0.17 | 0.22 | 0.45±0.16 | 0.40±0.18 | 0.50 |
| *Manual censoring* | 35±17 | 42±20 | 0.36 | 0.67±0.11 | 0.71±0.11 | 0.41 | 0.43±0.14 | 0.36±0.12 | 0.19 |
| *P*-values are calculated using an unpaired Student’s t-test and a *p*-value < 0.05 was considered statistically significant. Outcomes are based on the first scan of 15 patients and 10 controls. Manual censoring results are based on the first rating of rater 1. N_included_= number of included perforators; V_mean_= mean velocity during the cardiac cycle; PI= pulsatility index. | | | | | | | | | |

| 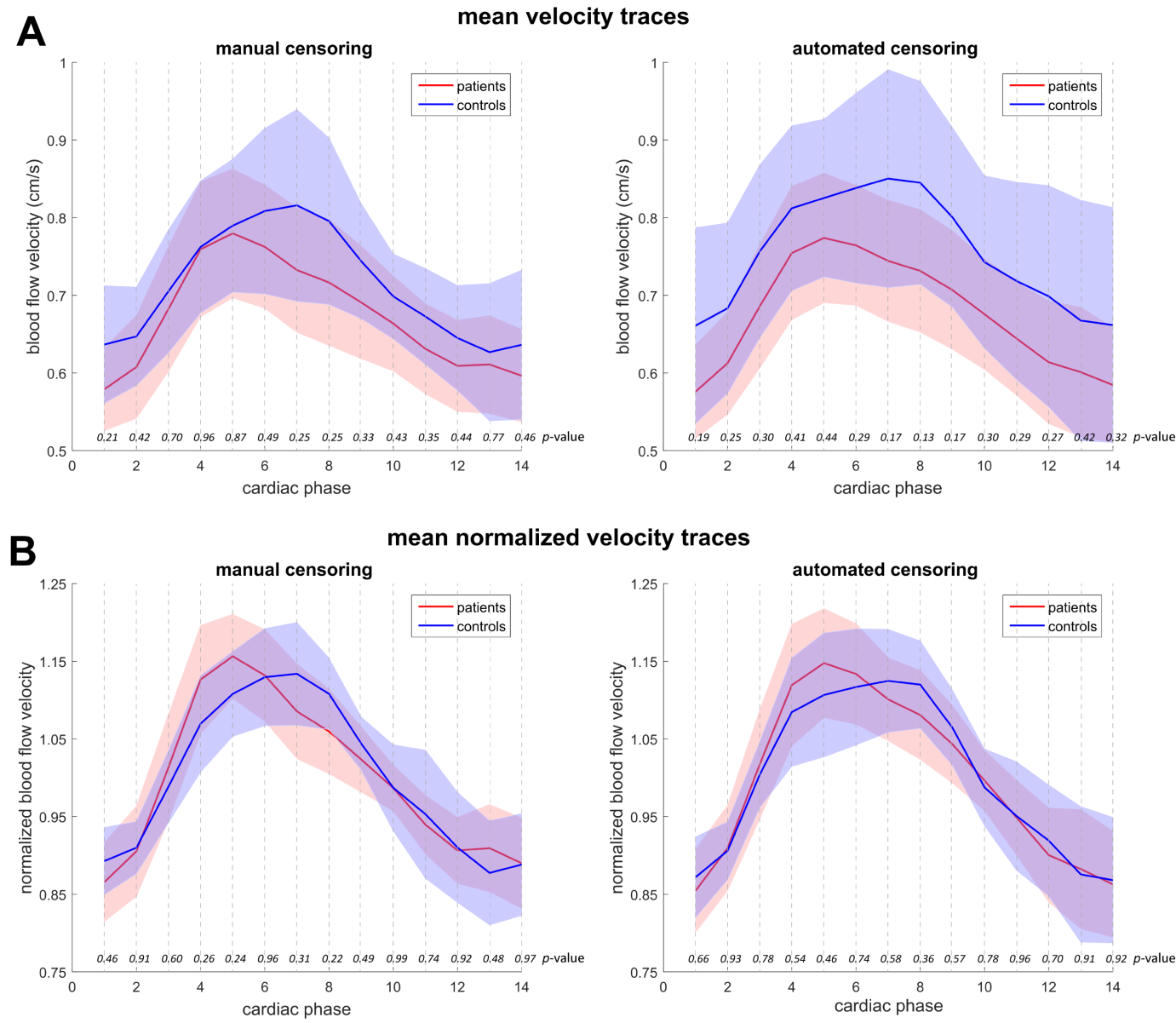 |
| --- |
| Figure A1: Comparison of the mean velocity traces (A) and mean normalized (division by the mean) velocity traces (B) between the patients (red curves) and controls (blue curves), for the manual censoring method (left) and the automated censoring method (right). Subject velocity traces are interpolated to the maximum number of occurring cardiac phases. Shaded regions indicate ± 2*standard error of the mean. Manual censoring results are based on the first rating of rater 1 at the first time point and the data used is based on scan 1. A statistically significant difference (*p* < 0.05) between patients and controls was tested for each cardiac phase using an independent Student’s t-test. *P*-values are given in italics. |

| 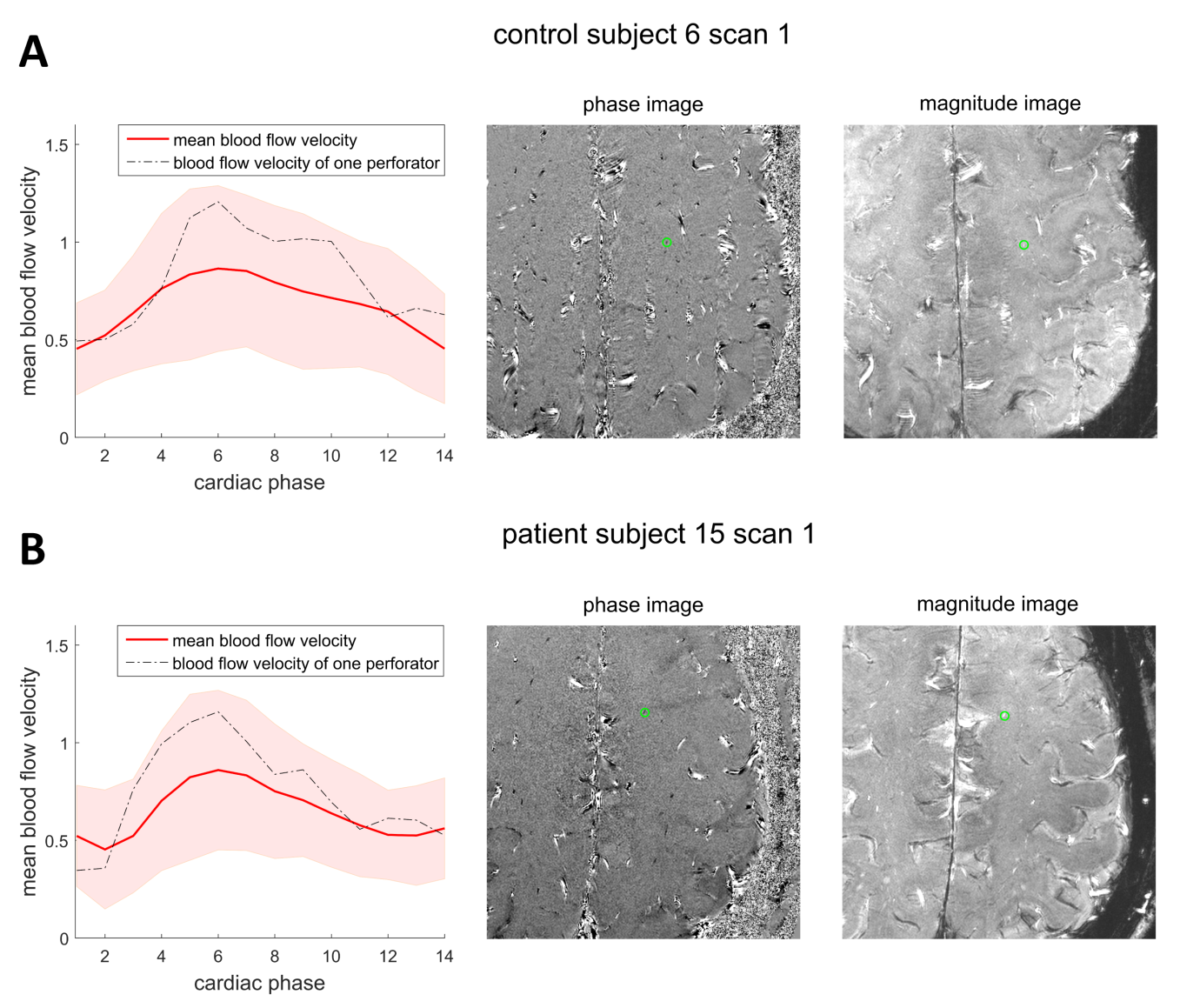 |
| --- |
| Figure A2: Mean velocity traces ± standard deviation of a representative subject from the control group (A) and a representative subject from the patient group (B). In addition, the trace of an individual perforator is given and the concerning perforator is circled in green on the phase and magnitude images of the 2D phase contrast scan (shown for cardiac phase #7). |

**Supplementary Material B**

| 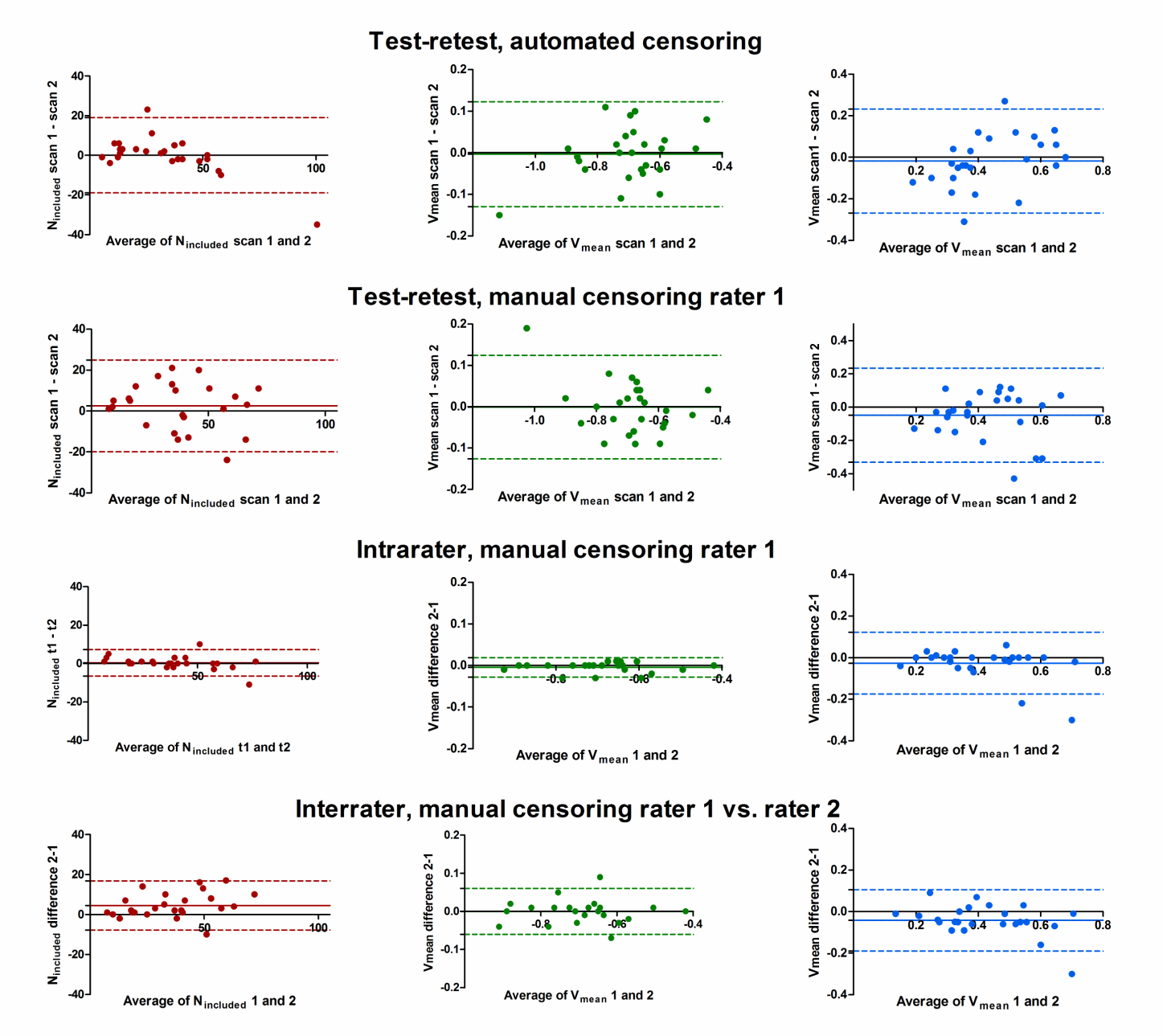 |
| --- |
| Figure B1: Bland-Altman plots showing the method reliability of the automated and the manual censoring method for test-retest, intrarater and interrater comparison. For the manual censoring method the interscan correlation of rater 1 and the correlation of rater 1 vs. rater 2 are based on the first of the repetitive scans. The test-retest correlation of rater 1 is based on both scans. Outcomes are based on 25 subjects. N_included_= number of included perforators; V_mean_= mean velocity over the cardiac cycle; PI= pulsatility index; t1= time point of first rating of rater 1, scan 1; t2= time point of second rating of rater 1, scan 1. |

**Supplementary Material C**

Two methods for calculating PI were compared:

Method 1: Averaging after PI calculation: Determine V_max_, V_min_ and V_mean_ of each detected perforator’s normalized velocity curve and calculate PI of each perforator. Averaging all PI’s results in a PI for each participant.

Method 2: Averaging before PI calculation: Average the normalized velocity curves of all perforators, and obtain V_max_, V_min_ and V_mean_ from this averaged curve to calculate a subject’s PI.

Monte Carlo simulations were performed to determine what measure of PI was least sensitive to noise, mimicking the velocity curve for 20 perforators and 15 cardiac phases. Fourteen SNR values ranging between 2 and 8.5 were used for the magnitude signal of the perforators. For all simulations, a single true value for V_mean_ and PI was used: V_mean_ = 1.0 cm/s and PI = 0.4. Thousand velocity simulations were performed, each with randomly assigned noise, σ_V_, where

σ_V_ = σ_phase_ ∙ (V_enc_/ᴨ) and σ_phase_ = 1/SNR_magnitude_

Simulation results for the two calculation methods for PI are depicted in Figure C1. For N=1 PI of both methods behaves equally as a function of magnitude SNR, since no averaging occurs. For N>1 for method 1, calculating PI of each perforator before averaging, will only narrow the 95% confidence interval, while its behavior with changing SNR remains the same (data not shown). For method 2, calculating PI from an average velocity curve, the overestimation of PI is greatly reduced in the entire SNR range when compared to method 1. Method 2 was therefore used for PI calculations in this study. The mean SNR±2*SD (5.8±2.5) of our data is also shown in Figure C1. This SNR is the SNR at the locations of the included perforators, averaged over all included perforators and all scans.

|  |
| --- |
| Figure C1: PI simulation results and standard deviations (SD), for various numbers of included perforators N for method 1 and method 2. The dotted horizontal line indicates the true PI value of 0.4. The mean SNR of the perforators selected in this study (5.8) is indicated by the vertical dotted line and its 2*SD range (3.25-8.27) is indicated by the grey filled area. On average, 33 (range: 5 - 118) vessels are detected with the automated method in this study. PI= pulsatility index. |

**Supplementary Material D**

Figure D1 shows that variation in the six parameters involved in the automated censoring method have only little impact on the outcome measures and the variation in the outcome measures remains within mean±2*standard error range.

| 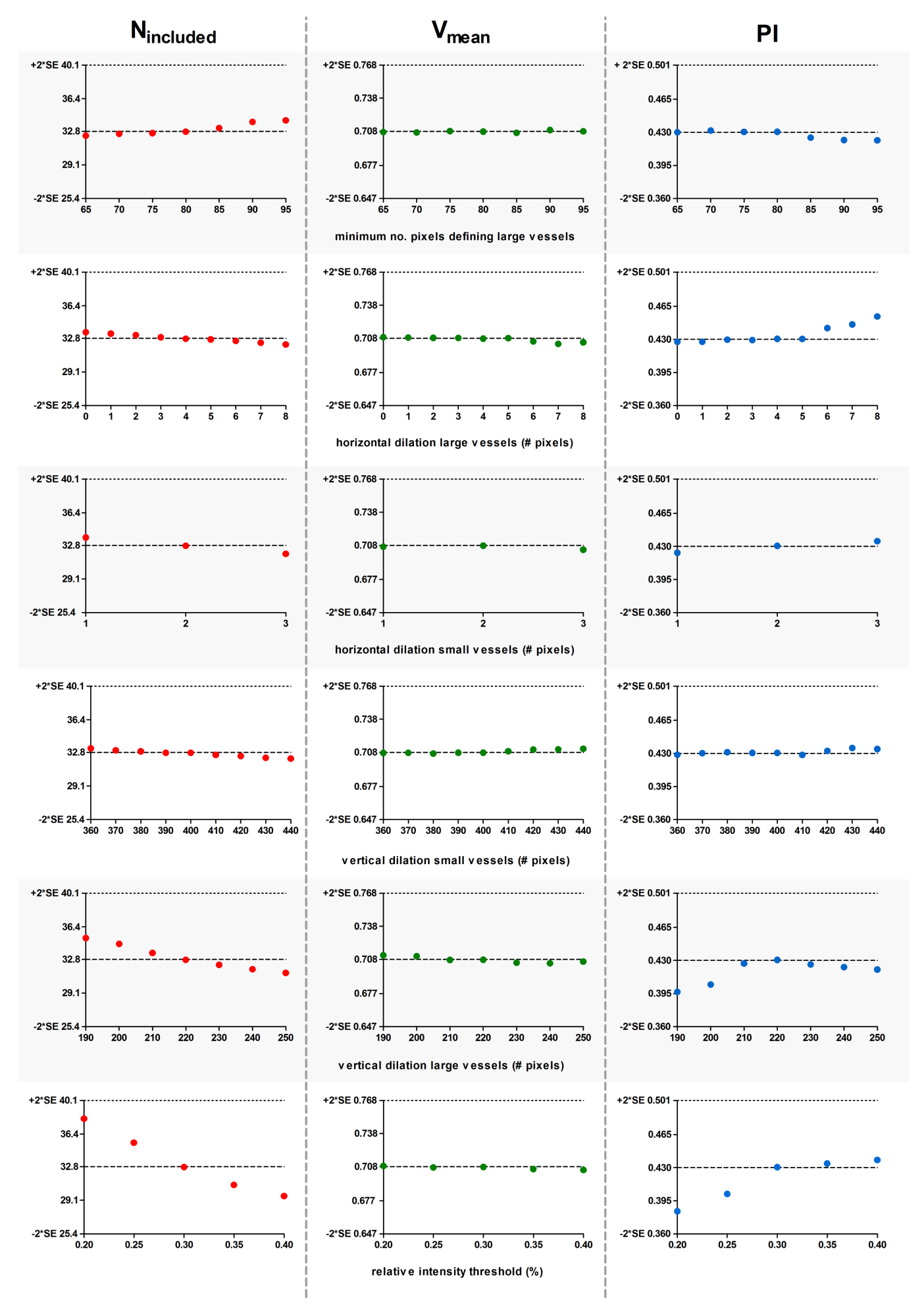 |
| --- |
| Figure D1: Plots showing the impact of variation in the six parameters involved in the automated censoring method on the outcome measures Nincluded, Vmean and PI. All results are based on the first of the repetitive scans. |
